# K_Ca_3.1 Channel-Blockade Attenuates Airway Pathophysiology in a Sheep Model of Chronic Asthma

**DOI:** 10.1371/journal.pone.0066886

**Published:** 2013-06-24

**Authors:** Joanne Van Der Velden, Grace Sum, Donna Barker, Emmanuel Koumoundouros, Garry Barcham, Heike Wulff, Neil Castle, Peter Bradding, Kenneth Snibson

**Affiliations:** 1 Faculty of Veterinary Science, The University of Melbourne, Parkville, Victoria, Australia; 2 Lung Health Research Centre, Department of Pharmacology, University of Melbourne, Parkville, Victoria, Australia; 3 Department of Electrical and Electronic Engineering, University of Melbourne, Parkville, Victoria, Australia; 4 Department of Pharmacology, University of California Davis, Davis, California, United States of America; 5 Icagen Inc., Durham, North Carolina, United States of America; 6 Department of Infection, Immunity and Inflammation, Institute for Lung Health, University of Leicester, Leicester, United Kingdom; University Hospital Freiburg, Germany

## Abstract

**Background:**

The Ca^2+^-activated K^+^ channel K_Ca_3.1 is expressed in several structural and inflammatory airway cell types and is proposed to play an important role in the pathophysiology of asthma. The aim of the current study was to determine whether inhibition of K_Ca_3.1 modifies experimental asthma in sheep.

**Methodology and Principal Findings:**

Atopic sheep were administered either 30 mg/kg Senicapoc (ICA-17073), a selective inhibitor of the K_Ca_3.1-channel, or vehicle alone (0.5% methylcellulose) twice daily (orally). Both groups received fortnightly aerosol challenges with house dust mite allergen for fourteen weeks. A separate sheep group received no allergen challenges or drug treatment. In the vehicle-control group, twelve weeks of allergen challenges resulted in a 60±19% increase in resting airway resistance, and this was completely attenuated by treatment with Senicapoc (0.25±12%; n = 10, P = 0.0147). The vehicle-control group had a peak-early phase increase in lung resistance of 82±21%, and this was reduced by 58% with Senicapoc treatment (24±14%; n = 10, P = 0.0288). Senicapoc-treated sheep also demonstrated reduced airway hyperresponsiveness, requiring a significantly higher dose of carbachol to increase resistance by 100% compared to allergen-challenged vehicle-control sheep (20±5 vs. 52±18 breath-units of carbachol; n = 10, P = 0.0340). Senicapoc also significantly reduced eosinophil numbers in bronchoalveolar lavage taken 48 hours post-allergen challenge, and reduced vascular remodelling.

**Conclusions:**

These findings suggest that K_Ca_3.1-activity contributes to allergen-induced airway responses, inflammation and vascular remodelling in a sheep model of asthma, and that inhibition of K_Ca_3.1 may be an effective strategy for blocking allergen-induced airway inflammation and hyperresponsiveness in humans.

## Introduction

Asthma affects 300 million people worldwide and is a significant cause of morbidity and mortality in developed countries. The financial burden of asthma in the United States is estimated to be over $10 billion. The disease is characterised by a complex of airway inflammation, remodelling and bronchial hyperresponsiveness, leading to recurring episodes of potentially reversible airflow limitation and in some patients fixed airflow obstruction. Currently available therapies for the treatment of asthma are ineffective at relieving symptoms in approximately 10% of patients and offer no prospect of cure. These patients with apparently refractory disease have significant morbidity and mortality, and there is therefore an urgent need to identify better therapeutic approaches to treat asthma.

Ion channels are emerging as interesting therapeutic targets for the management of asthma, particularly ion cannels that directly or indirectly facilitate the entry of calcium ions (Ca^2+^) into cells [1]. The K_Ca_3.1 ion-channel is expressed by a wide variety of cells involved in asthma including mast cells, T cells, airway epithelial cells, fibroblasts, fibrocytes, and dedifferentiated, proliferative airway smooth muscle cells [1], [2]. Many of the complex cellular processes mediated by these cells require a sustained elevation of intracellular Ca^2+^, including cellular proliferation, chemotaxis, mediator-release and for smooth muscle, contraction [3]. As the influx of positively charged Ca^2+^ ions depolarises the cell membrane, the driving force for Ca^2+^ entry soon diminishes. However, rising cytoplasmic Ca^2+^ levels activate the Ca^2+^ activated K^+^ channel K_Ca_3.1 resulting in K^+^ efflux, which maintains a negative membrane potential and sustains a driving force for Ca^2+^ entry [4], [5]. Modulation of the K_Ca_3.1 channel, could therefore potentially limit the activity of several structural and inflammatory cells that play important roles in disease pathophysiology and lead to the development of new therapies for the treatment of asthma [1].

To date, studies investigating the effects of K_Ca_3.1 blockade in relation to asthma have been encouraging. Studies have demonstrated that blockade of K_Ca_3.1 inhibits ASM cell proliferation *in vitro* [2] and attenuates the chemotactic response of human lung mast cells (HLMCs) to conditioned media from asthmatic ASM [6]. Studies have also shown that allergen-induced mast cell degranulation and IgE-mediated systemic anaphylactic responses are attenuated in mice lacking the K_Ca_3.1 channel [7]. While these studies indicate that modulation of the K_Ca_3.1 ion-channel could be of potential therapeutic benefit in asthma, the effect of K_Ca_3.1 blockade on airway remodelling, inflammatory responses and functional airway responses *in vivo* is largely unknown.

In the present study we investigate the effect of K_Ca_3.1-inhibition, on *in vivo* airway responses and inflammation in a large animal model of asthma. To achieve this, house dust mite (HDM) sensitised sheep were treated twice daily with Senicapoc (ICA-17073) or vehicle and challenged biweekly with HDM. We examined the effect of K_Ca_3.1-blockade on resting lung function and airway responses to allergic and non-allergic stimuli. We also studied the effect of this treatment on allergen-induced airway remodelling and inflammatory responses in these sheep.

## Methods

### Experimental Animals and Allergen Sensitisation

Female Merino-cross sheep (6 months) were immunised with HDM (50 µg/mL; *Dermatophagoides pteronyssins*; CSL, Parkville, Australia) and atopic sheep selected as described previously [8]. All experimental animal procedures and the collection of tissues/cells were approved by the Animal Experimentation Ethics Committee of the University of Melbourne.

### Treatment

Sheep received either fortnightly challenges with HDM or no treatment for fourteen weeks (n = 10 per group). For HDM challenges sheep were ventilated (BEAR 2; Bear Medical Systems, Palm Springs, USA) at 20 breaths per minute with a nebulised 1 mg/mL HDM solution for 10 minutes. HDM-challenged sheep received twice daily oral dosing with a syringe of either 30 mg/kg Senicapoc (Icagen Inc; Durham, USA) in vehicle, 0.5% Methylcel® MC (Sigma-Aldrich, Castle Hill, Australia) in distilled water, or vehicle alone. Dosing commenced seven days prior to the first HDM challenge and continued throughout the trial.

### Lung Function

Measurement of lung function and airway responsiveness to carbachol was assessed as previously described [9]. Airway responsiveness is expressed as the concentration of carbachol aerosol (1 breath unit [BU] is 1 breath of 1% *w/v* carbachol) needed to increase lung resistance (R_L_) by 100% from baseline.

### Bronchoalveolar Lavage

Bronchoalveolar lavage (BAL) cells were collected from sheep before challenge and again at 48 hours after a HDM aerosol challenge, four weeks into the repetitive challenge regime as previously described [10]. Total and differential leukocyte counts were performed as previously described [10].

### Lung Tissue Collection, Immunohistochemistry and Morphometric Analysis

Seven days after the final HDM challenge, sheep were euthanised by intravenous barbiturate overdose (Lethabarb). Lung segments from the left and right caudal lungs were removed and inflated with optimal cutting temperature compound (ProSciTech, Thuringoura, Australia) diluted in phosphate buffered saline (1∶1). Mast cells, eosinophils and blood vessels were identified immunohistochemically as previously described [11], [12]. Mouse monoclonal antibody supernatants against ovine cell types were used to identify T-lymphocyte subpopulations: CD8, CD4, CD45, CD45R and gammadelta (Centre for Animal Biotechnology). HRP-conjugated secondary antibodies (Dako) and a peroxidase-based detection system were used for visualization. Specificity of staining was confirmed by omission of the primary antibody. Collagen and ASM was stained and measured on sections as previously described [11].

All morphometric measurements of the airways were made using digital image analysis (Image-Pro Plus, Media Cybernetics, version 4.1.0.0) by a single observer blinded to the experimental group as previously described [11], [12]. Measurements were made on two small cartilaginous bronchi, approximately 1–2 mm diameter, from each sheep and the two values averaged.

### Statistical Analysis

All values are reported as mean ± SEM. Data were analysed using GraphPad Prism 5.0 statistical software (GraphPad Software Inc., La Jolla, USA). Comparisons between different time-points within one group were analysed with a Wilcoxon-signed rank test. Comparisons between the vehicle and Senicapoc group were performed using a Mann-Whitney test. A one-way ANOVA was used to compare between three groups. A P value of <0.05 was taken as significant.

## Results

### Lung Function

**Figure 1:**
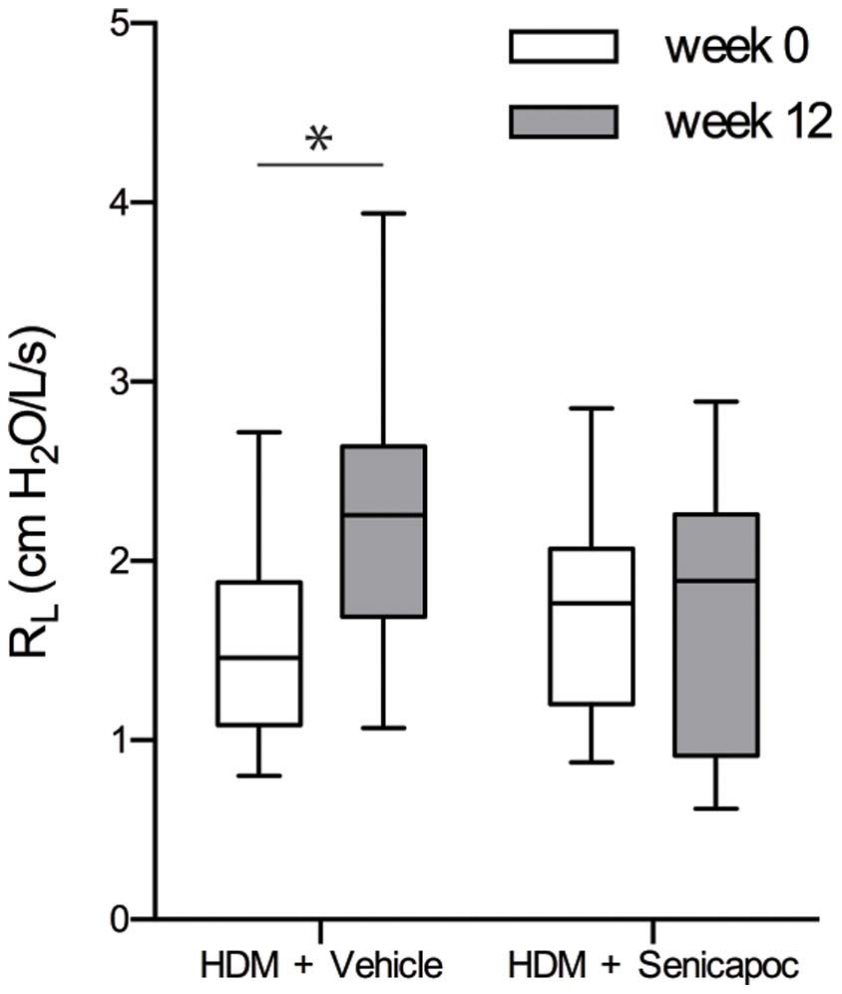
Comparison of resting lung resistance (R_L_) as assessed prior to the first HDM challenge (week 0) and 12 weeks into the repeated challenge regime in sheep treated with either vehicle or Senicapoc. Horizontal lines represent the median, boxes represent the 25^th^ and 75^th^ percentiles, whiskers represent the 5^th^ and 95^th^ percentiles. n = 10, **P<0.01.

There was an increase of 60±20% between week 0 and week 12 in the resting lung resistance (R_L_) in HDM-challenged sheep that received vehicle alone (P<0.05). An increase in R_L_ was inhibited completely by treatment with Senicapoc (0.25±12%; Fig. 1).

**Figure 2:**
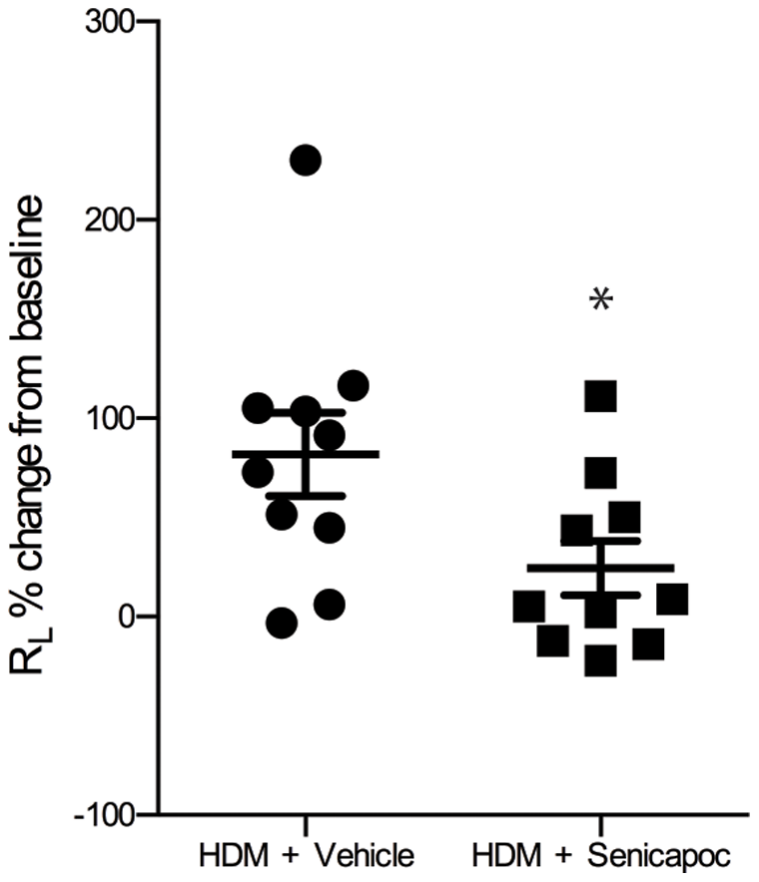
The percent acute change in lung resistance (R_L_) following HDM challenge as assessed at week 12 in sheep treated with either vehicle or Senicapoc. Dots represent data points for individual sheep, lines show mean ± SEM. n = 10, *P<0.05.

**Figure 3:**
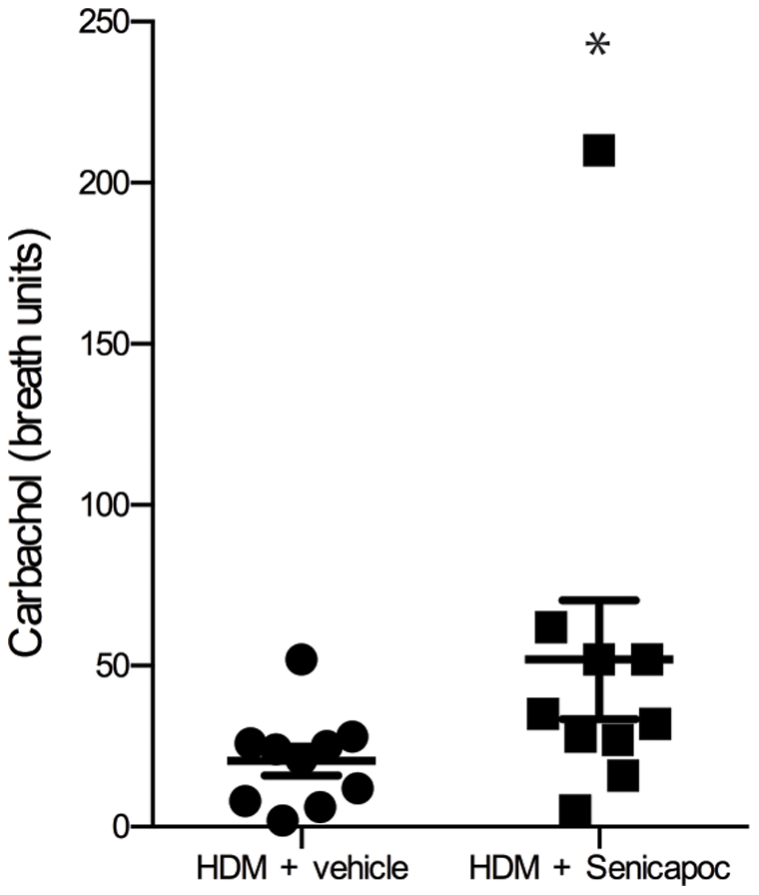
Comparison of the number of breath units (BU) of carbachol required to raise the lung resistance (R_L_) 100% above the R_L_ following the administration of saline (1 BU is equal to 1 breath of 1% carbachol). Dots represent data points for individual sheep, lines show mean ± SEM. n = 10, *P<0.05.

The peak early phase bronchoconstriction response following HDM challenge was reduced by 58% in Senicapoc treated sheep compared to sheep treated with vehicle alone (P<0.05; Fig. 2). There was no pronounced late-phase response detected 5 or 24 hours following HDM-challenge in either the vehicle or Senicapoc-treated sheep (data not shown). Senicapoc treatment also resulted in a reduction in airway responsiveness to the cholinergic agonist carbachol compared with vehicle 24-hours post-HDM challenge. Sheep treated with Senicapoc required a significantly higher dose of carbachol, (measured as an increase in breath units (BU) of carbachol delivered), to increase R_L_ by 100% compared to sheep treated with vehicle (52±18 vs. 20±5; Fig. 3).

### Bronchoalveolar Lavage

Table 1 shows the total and differential cell counts on BAL cells collected prior to, and 48 hours following, the 4^th^ HDM challenge for both the vehicle and Senicapoc-treated sheep at Week 4 of the fortnightly allergen challenge regime. Immediately before the 4^th^ HDM challenge (i.e. 7 days post 3^rd^ HDM challenge), there were significantly fewer neutrophils and lymphocytes in the resting BAL of Senicapoc-treated sheep compared to vehicle-treated sheep (P<0.05).

**Table 1:** Total and differential leukocyte counts were performed on BAL cells collected from vehicle and Senicapoc treated sheep prior to and 48 hours following the house dust mite (HDM) challenge, at week 4.

	vehicle	Senicapoc
	Pre-HDM challenge	
Total leukocytes	35.6±0.5	20.1±0.3
Neutrophils	2.7±0.9	0.7±0.9*
Eosinophils	1.9±0.9	2.4±0.8
Lymphocytes	4.3±1.0	1.7±0.2*
Macrophages	26.3±6.8	15.0±2.2
	48 hrs post-HDM challenge	
Total leukocytes	48.0±0.8	42.0±1.0§§
Neutrophils	4.5±1.3	8.3±3.0§
Eosinophils	16.4±4.9§§	4.1±1.1*
Lymphocytes	3.6±1.3§	4.0±1.3§
Macrophages	22.8±3.0§§	25.5±5.0§

All values shown are ×10^4^ cells per mL BAL fluid. Values are the mean ± SEM. n = 10,

* P<0.05,

** P<0.01 compared to vehicle.

§ P<0.05,

§§ P<0.01 compared to pre-HDM challenge.

The differential counts at 48 hours after the 4^th^ HDM challenge showed a rise in BAL eosinophils in the vehicle-treated group, and this was markedly attenuated by Senicapoc treatment (P<0.05). Interestingly, treatment with Senicapoc did not affect the total leukocyte recruitment in BAL 48 hours following a HDM challenge compared to vehicle. There were no differences between the numbers of neutrophils, lymphocytes or macrophages in retrieved in the BAL between any of the groups 48 hours following HDM challenge (Table 1).

### Eosinophil Density

**Figure 4:**
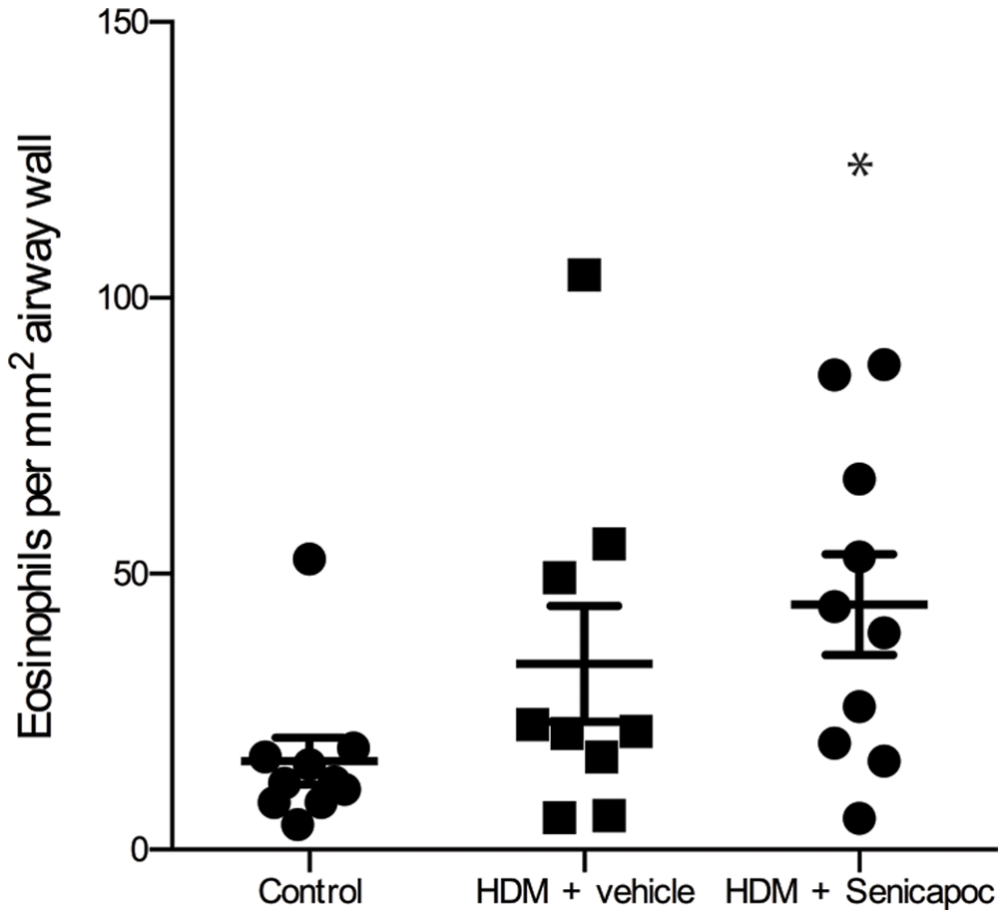
Eosinophils per mm^2^ airway wall in control sheep and HDM challenged sheep treated with either vehicle or Senicapoc. Dots represent data points for individual sheep, lines show mean ± SEM. n = 10, *P<0.05 compared to control.

Airway tissue collected post-mortem, for both the Senicapoc- and vehicle-treated HDM-challenged groups there were greater than 2-fold increases in eosinophil densities compared to untreated controls (Fig. 4) (control 16±4 cells/mm^2^, vehicle 36±29 cells/mm^2^, Senicapoc 44±9 cells/mm^2^, P<0.05).

### Mast Cell Density

**Figure 5:**
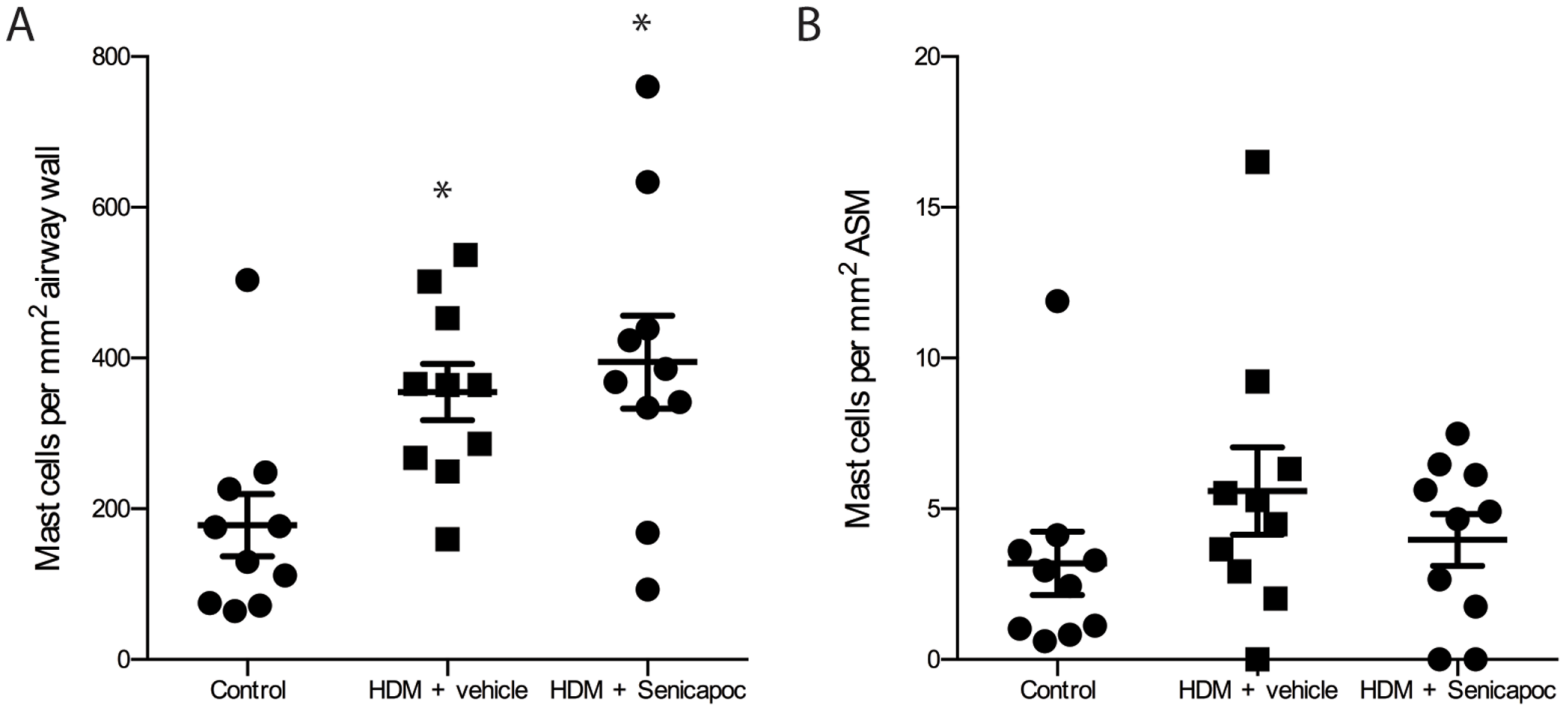
Mast cells per mm^2^ A) airway wall and B) airway smooth muscle (ASM) in control sheep and HDM-challenged sheep treated either with vehicle or Senicapoc. Dots represent data points for individual sheep, lines show mean ± SEM. n = 10, *P<0.05 compared to control.

In airway tissue collected post-mortem, mast cell density in the airway wall was significantly increased in vehicle-treated HDM-challenged sheep compared to untreated controls (355±37 vs. 178±42 cells/mm^2^, P<0.05; Fig. 5a). Mast cell density was also higher in the airway walls of Senicapoc-treated HDM-challenged sheep compared with controls (395±62 vs. 178±42 cells/mm^2^, P<0.05; Fig. 5a). There were no significant differences in the number of mast cells within the ASM bundles between any of the groups (control: 3.2±1.0, vehicle: 5.6±1.4, Senicapoc: 3.9±0.9 cells/mm^2^, n = 10, P = 0.265).

### Airway Remodelling

**Figure 6:**
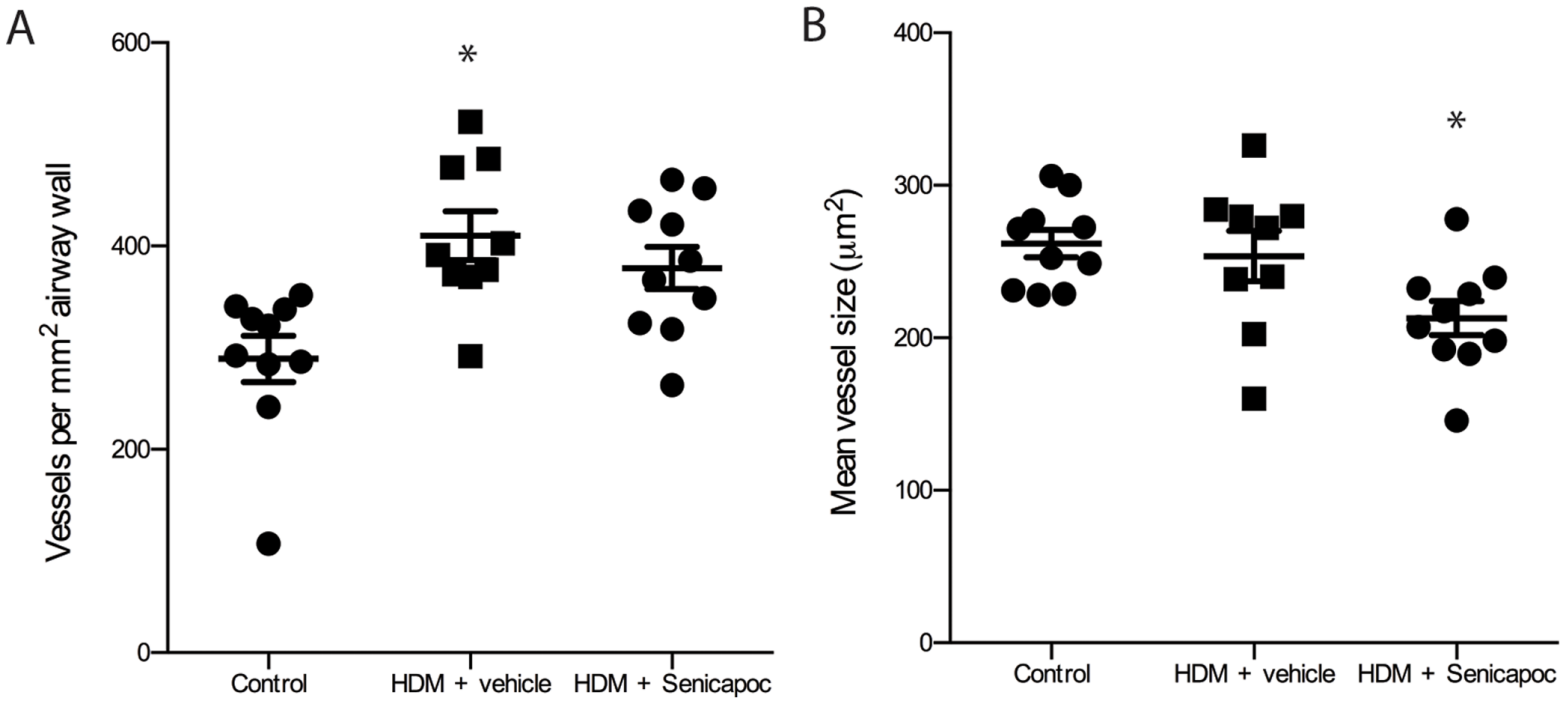
Blood vessel density and mean blood vessel size. **A**) vessels per mm^2^ airway wall **B**) mean blood vessel size (µm^2^) in control sheep and HDM-challenged sheep treated wither with vehicle or Senicapoc. Dots represent data points for individual sheep, lines show mean ± SEM. n = 10, *P<0.05 compared to control.

There was a significant increase in the density of blood vessels in the airway wall in HDM-challenged sheep treated with vehicle compared to control sheep (410±24 vs. 289±23 vessels/mm^2^, P<0.05; Fig. 6a). Blood vessel density was also increased in the Senicapoc-treated group of sheep but not significantly different from either vehicle, or control groups (378±21 vs. 289±23 vessels/mm^2^, P = 0.312, Fig. 6a). Blood vessel size was similar in control sheep and vehicle-treated HDM-challenged sheep, but significantly decreased in Senicapoc-treated HDM-challenged animals compared to controls (213±11 vs. 261±9 µm^2^ respectively, P<0.05; Fig. 6b). There was no difference in ASM or collagen area per mm^2^ basement membrane between any of the groups (data not shown).

### T-lymphocyte Subpopulations

**Figure 7:**
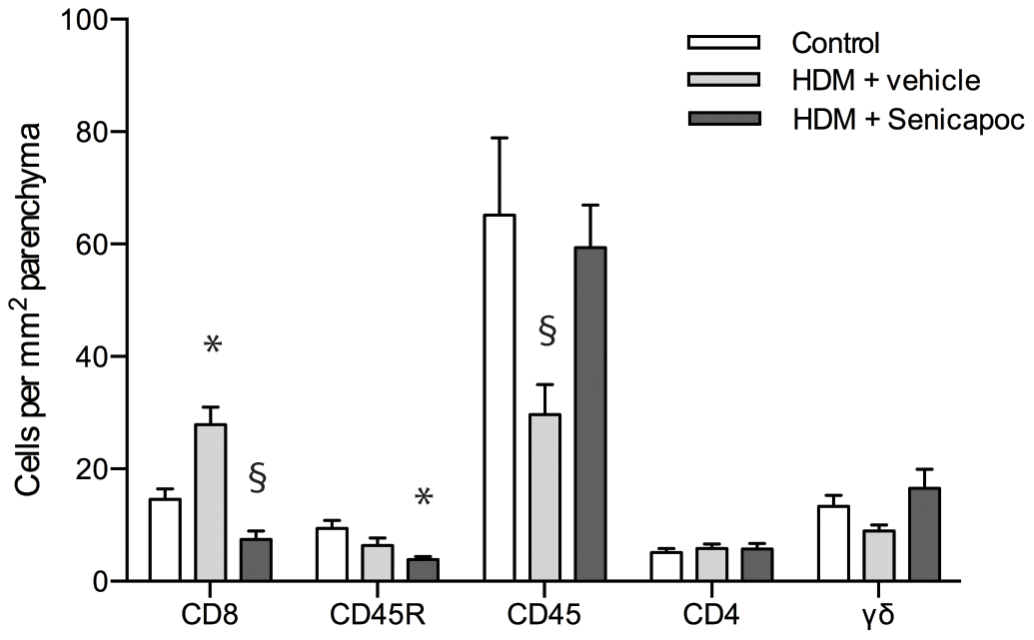
T-lymphocyte subpopulations. **Cells per mm^2^ parenchyma for control sheep and HDM challenged sheep treated with either vehicle or Senicapoc.** Mean+SEM. n = 10, *P<0.05 compared to control, ^§^P<0.05 compared to vehicle.

The number of CD8^+^ cells per mm^2^ in lung parenchyma was significantly increased in the vehicle-treated HDM-challenged group compared to unchallenged controls (29±3 vs. 15±2 cells/mm^2^, P<0.05; Fig. 7). In contrast, in Senicapoc-treated sheep, the density of CD8+ lymphocytes was significantly lower than that found for both the HDM-challenged vehicle-treated sheep (7±2 vs. 29±3 cells/mm^2^ P<0.05), and control sheep (7±2 vs. 15±2 cells/mm^2^ P<0.05). The concentration of CD45^+^ cells was decreased in vehicle-treated HDM-challenged sheep compared to controls (30±5 vs. 65±14 cells/mm^2^, P<0.05), while for this lymphocyte subset there were no difference between Senicapoc-treated and control sheep (59±8 vs. 65±14 cells/mm^2^). There was a significant decrease in the density of CD45R^+^ cells in the Senicapoc-treated sheep compared to controls (4±1 vs. 9±3 cells/mm^2^, P<0.05; Fig. 7). There were no significant differences in the densities of CD4 and gammadelta positive-cells between any of the groups (Fig. 7).

## Discussion

The K_Ca_3.1-channel is expressed by several structural and inflammatory cells in the airways and is considered to play a significant role in driving pathogenic changes associated with asthma [1], [2]. While to date much of the evidence supporting the importance of K_Ca_3.1-activity in allergic airways disease comes from *in vitro* studies, results from the present study provide support for the hypothesis that K_Ca_3.1 activity plays an important role in the generation of allergen-induced airway responses *in vivo*. In particular, we have shown that K_Ca_3.1-blockade via oral Senicapoc administration was able to significantly reduce acute allergen-dependent bronchoconstriction, the allergen-dependent increase in lung resistance, and airway hyperresponsiveness (AHR) to carbachol.

From previous work we would predict that the beneficial effects observed with Senicapoc treatment with respect to lung physiology would occur in part through the attenuation of mast cell activity. Our study found no difference in the number of mast cells present between the Senicapoc- and vehicle-treated sheep. This finding is in line with previous studies, which demonstrate that K_Ca_3.1^−/−^ mice have normal mast cell numbers compared to wild-type mice and that pharmacological inhibition of K_Ca_3.1 does not attenuate the proliferation of HLMCs *in vitro* [6], [7]. It has previously been reported that mast cell-degranulation is inhibited in mice that lack the K_Ca_3.1 channel, and in HLMCs exposed to K_Ca_3.1 blockers [7], [13]. Attenuated mast-cell degranulation could therefore potentially explain our observation that K_Ca_3.1-blockade significantly reduced the peak early-phase response to HDM, as this response in sheep is a mast cell-dependent process [14].

The K_Ca_3.1-channel is also expressed by both asthmatic and non-asthmatic ASM, and its inhibition attenuates human ASM proliferation *in vitro* [2]. It is therefore possible that long term blockade of K_Ca_3.1 is able to prevent increases in the amount of ASM in asthmatic airways. Previous studies using the sheep model have shown that 24–26 weeks of HDM challenge is sufficient to induce airway-remodelling changes typical of asthma including increases in airway collagen and ASM content [11], [12]. However, in the present study neither of these remodelling changes was observed, suggesting that 14 weeks of HDM challenges was not sufficient to induce structural airway changes. Thus, we are unable to determine whether K_Ca_3.1 blockade has any effect on structural airway remodelling in asthma.

Blockade of the K_Ca_3.1 channel block significantly reduced the numbers of eosinophils in the BAL following allergen-challenge. Surprisingly, treatment with Senicapoc had no effect on the density of eosinophils in the airway tissue. K_Ca_3.1-activity is required for human mast cell and fibrocyte migration, and it possible that this is also the case for eosinophil migration [6], [15]. This is similar to reports of anti-IL-5 treatment, which markedly reduces sputum eosinophils by is less effective at reducing tissue eosinophils [16], [17]. Furthermore, anti-IL-5 treatment has been shown to be effective at reducing exacerbations in patients with severe eosinophilic asthma [18]. This raises the possibility that Senicapoc may have similar effects in this group of patients, and could offer an alternative therapeutic option for patients with relative steroid resistance.

Interestingly, while there was a significant increase in neutrophils 48 hours post-HDM-challenge in both the vehicle and Senicapoc treated groups, the magnitude of the increase was ten-fold higher in the Senicapoc treated group. Most of this change was due to the significant reduction in the number of neutrophils per mL in the Senicapoc group, compared to the vehicle treated group, immediately prior to HDM challenge.

Endothelial cell proliferation and migration, which gives rise to the formation of new blood vessels, is induced by an elevation in angiogenic factors such as basic fibroblast growth factor (bFGF) and vascular endothelial growth factor (VEGF) and is initiated by a rise in intracellular Ca^2+^ [19]. Vascular remodeling is a feature of asthma, and is inversely associated with the post-bronchodilator FEV_1_ but not AHR [20]. In an *in vivo* mouse model of angiogenesis K_Ca_3.1-blockade via daily administration of TRAM-34, another selective K_Ca_3.1 channel blocker significantly reduced bFGF- and VEGF-induced endothelial cell proliferation and angiogenesis [21]. Our current results indicate that K_Ca_3.1-blockade also dampens an increase in vessel density in an *in vivo* model of allergic airways disease. There is currently no published data on the effect of K_Ca_3.1-blockade on blood vessel size. However, the results presented here show a significant reduction in the mean size of vessels in the airway wall of Senicapoc-treated sheep. It is possible that this reduced vessel calibre contributes to a reduction in airway oedema in the airways following allergen exposure, and thus accounts for some of the observed improvements in lung function.

In asthmatic airways, T cells are believed to contribute to disease pathophysiology through the release of a plethora of cytokines that are involved in the induction of airway inflammation. In the present study we found a reduction in the number of CD8 and CD45R positive T cells in the airway parenchyma of Senicapoc-treated sheep compared to untreated controls and/or vehicle treated sheep. This is in line with previous studies which have demonstrated that K_Ca_3.1-blockade via charybdotoxin, clotrimazole and TRAM-34 have been shown to inhibit T cell proliferation [22] as well as cytokine secretion *in vitro* [23]. However, the role, if any, for CD8^+^ T cells in asthma is uncertain [24].

Targeting K_Ca_3.1 is an attractive therapeutic target, as its inhibition does not appear to have adverse effects on healthy physiology. The K_Ca_3.1-knockout mouse has a relatively healthy phenotype, long-term treatment of animals with TRAM-34 has not produced any severe adverse effects, and Senicapoc was well tolerated in phase III trials of sickle cell disease when given for 48 weeks [4]. In a small phase II trial in patients with allergic asthma, 2 weeks treatment with Senicapoc attenuated the late-phase response to allergen challenge and reduced the concentration of exhaled nitric oxide [4].

In summary, this study has shown that the prolonged inhibition of the K_Ca_3.1 channel by oral Senicapoc administration is effective at reducing the severity of allergen-induced early-phase bronchoconstriction, resting lung resistance, AHR, BAL eosinophilia and vascular remodelling in this sheep model of experimental asthma. These findings support the hypothesis that the K_Ca_3.1 channel plays an important role in asthma pathophysiology and indicate that targeting this channel for several months could be an effective and novel therapeutic strategy.
